# Defining Memory CD8 T Cell

**DOI:** 10.3389/fimmu.2018.02692

**Published:** 2018-11-20

**Authors:** Matthew D. Martin, Vladimir P. Badovinac

**Affiliations:** ^1^Department of Pathology, University of Iowa, Iowa City, IA, United States; ^2^Department of Microbiology and Immunology, University of Iowa, Iowa City, IA, United States; ^3^Interdisciplinary Graduate Program in Immunology, University of Iowa, Iowa City, IA, United States

**Keywords:** CD8 T cell, memory, subsets, heterogeneity, protection, outbred mice, age of memory, history of Ag enounters

## Abstract

CD8 T cells comprising the memory pool display considerable heterogeneity, with individual cells differing in phenotype and function. This review will focus on our current understanding of heterogeneity within the antigen-specific memory CD8 T cell compartment and classifications of memory CD8 T cell subsets with defined and discrete functionalities. Recent data suggest that phenotype and/or function of numerically stable circulatory memory CD8 T cells are defined by the age of memory CD8 T cell (or time after initial antigen-encounter). In addition, history of antigen stimulations has a profound effect on memory CD8 T cell populations, suggesting that repeated infections (or vaccination) have the capacity to further shape the memory CD8 T cell pool. Finally, genetic background of hosts and history of exposure to diverse microorganisms likely contribute to the observed heterogeneity in the memory CD8 T cell compartment. Extending our tool box and exploring alternative mouse models (i.e., “dirty” and/or outbred mice) to encompass and better model diversity observed in humans will remain an important goal for the near future that will likely shed new light into the mechanisms that govern biology of memory CD8 T cells.

## Introduction

At the most basic level, a memory CD8 T cell can be defined as a CD8 T cell that has responded to cognate antigen (Ag) and persists long-term. Such a simple definition does little to account for immune-mediated protection, however, and hosts possessing memory CD8 T cells are often better protected against solid tumors and infection with intracellular bacteria, viruses, and protozoan parasites than their naïve counterparts (1–8). To encompass protective capabilities, our definition would need to expand to include quantitative and qualitative aspects of CD8 T cell memory and how these differ from naïve and effector CD8 T cells. Compared to naïve cells of the same antigen-specificity, memory CD8 T cells persist in greater numbers (9); can populate peripheral organs (10); are poised to immediately proliferate, execute cytotoxic functions, and secrete effector cytokines upon Ag re-encounter (11–16); and exist in different metabolic, transcriptional, and epigenetic states (17–20). Despite some similarities between effector and memory CD8 T cells at the molecular, epigenetic, metabolic, and functional levels (17–23), memory cells persist long-term while effector cells undergo robust contraction (18, 24), and unlike effector cells they are capable of vigorous proliferation following Ag re-encounter (25).

Expanding our definition to account for functional differences between naïve, effector, and memory cells helps to clarify why immune hosts are better protected than naïve hosts, but it does not begin to explain why some memory CD8 T cell responses are more protective than others. While a correlation between the numbers of memory CD8 T cells and the level of protection is firmly established (26, 27), quality (or functional ability) of memory CD8 T cells also determines the degree of memory CD8 T cell-mediated protection. Characteristics of memory CD8 T cell responses best-suited to provide protection against infection vary depending upon the nature of the pathogen, and over the past 20 years it has become clear that the memory CD8 T cell pool consists of a heterogeneous population of cells that differ in phenotype, function, and protective capacity (28–34). A complete definition of CD8 T cell memory, then, should account for this diversity, and immunologists have categorized cells of distinct functional abilities into subsets to better understand memory CD8 T cell heterogeneity. Heterogeneity within memory CD8 T cell subsets uncovered by recent subsetting strategies discussed in this review also highlights the limitations of ascribing discrete functions to memory CD8 T cells expressing one or two phenotypic markers. However, despite these limitations, current subset classifications do provide valuable predictive information on the likelihood that cells of a given phenotype will be able to perform a defined function in response to a particular pathogen.

CD8 T cells of memory phenotype and function can arise in response to self-peptide and/or in a lymphopenic environment in response to cytokines that trigger homeostatic proliferation (“virtual” and “innate” memory) (35, 36). However, this review will focus solely on memory CD8 T cells generated in response to infection. Specifically, we will discuss (1) current subset classifications of memory CD8 T cells, (2) how subset composition is shaped following time after infection and upon additional Ag encounters, (3) how memory CD8 T cell subsets in humans compare to those in mice, and (4) how mouse studies that better model human biology inform our knowledge of memory CD8 T cell biology.

## Memory CD8T cell subsets

### Effector (Tem) and central (Tcm) memory

Although the number of memory CD8 T cell subsets has expanded (Table 1), the first characterization of heterogeneity within a memory CD8 T cell pool of undefined origin in humans described two subsets—CD62L^lo^/CCR7^lo^ effector memory (Tem) and CD62L^hi^/CCR7^hi^ central memory (Tcm) cells (37). Expression of CCR7 and CD62L on Tcm cells facilitates homing to secondary lymphoid organs, while Tem cells are more cytolytic and express integrins and chemokine receptors necessary for localization to inflamed tissues (37). This description led to the paradigm that the memory CD8 T cell population consists of specialized cells that uniquely participate in the immune response to confer host protection. Mechanistic studies in mice showed that Tem cells were more prevalent in tissues, while Tcm cells were more prevalent in lymph nodes and better equipped to persist following infection and to produce IL-2 and proliferate in response to Ag (30). Transcription factors promote the development and function of Tem and Tcm cells, and T-bet, Blimp1, ID2, and STAT4 expression is associated with Tem cells, while Eomes, TCF1, BCL-6, ID3, and STAT3 expression is associated with Tcm cells (38–42, 45–42). Tcm cells provide enhanced protection against chronic infection with LCMV-clone 13 (30), while Tem cells provide superior protection against infection with vaccinia virus, and in some instances *Listeria monocytogenes* (31, 63). These studies led to the hypothesis that Tcm cells are specialized to handle systemic infections due to their centralized location within secondary lymphoid organs and superior proliferative abilities, and that Tem are specialized to handle infections arising within peripheral organs due to their cytotoxicity and ability to localize to tissues.

**Table 1:** Memory CD8 T cell subsets.

**Subset**	**Phenotype**	**Function**	**Location/Trafficking**	**Transcription factors**	**References**
Tem	CCR7^lo^/CD62L^lo^ Cx3Cr1^hi^/CD27^lo^ CD127^hi^ CD27^−^/CD45RA^−^ (humans)	++ Cytotoxicity +- Proliferation	Circulation	Tbet^hi^ Blimp1^hi^/ID2^hi^/STAT4^hi^	(30, 37–44)
Tcm	CCR7^hi^/CD62L^hi^ Cx3Cr1^lo^/CD27^hi^ CD127^hi^ CD27^+^/CD45RA^−^ (humans)	+− Cytotoxicity ++ Proliferation	Circulation Lymph nodes	Tbet^lo^ Eomes^hi^/TCF1^hi^/Bcl6^hi^/ STAT3^hi^/ID3^hi^	(30, 37–39, 42–49)
Temra (humans)	CCR7^−^/CD27^−^/CD45RA^+^ CD127^lo^	++ Cytotoxicity +− Proliferation	Circulation		(44)
Trm	CD69^hi^/CD103^hi^/CD49a^hi^ (depending on tissue) CXCR3^hi^/KLRG1^lo^/CCR7^lo^/ CD62L^lo^, CD127^hi^ Cx3Cr1^lo/int^	Sensing and alarm + proliferation	Tissue resident	KLF2^−/lo^/Eomes^−/lo^ Tbet^lo^/TCF1^lo^ Hobit^hi^/Blimp1^hi^	(33, 43, 50–60)
Tpm	CCR7^+/−^/CD62L^+/−^/CD127^hi^ Cx3Cr1^int^/CD27^hi^	+ Cytotoxicity + Proliferation	Circulation Tissue trafficking Lymph nodes	Tbet^+/−^	(43, 61, 62)
Others	CD27^lo^/CD43^lo^ KLRG1^hi^, CD127^lo^	++ Cytotoxicity +− Proliferation	Tissue trafficking	Tbet^hi^/Eomes^lo^	(32, 62)

With identification of memory subsets came questions of when CD8 T cells of discrete function form during a response and how effector cells survive to populate the heterogeneous memory CD8 T cell pool. Interleukin 7 is required for the survival of naïve cells and promotes the survival of memory CD8 T cells (64), and initial reports suggested that the expression of CD127, the alpha chain of the IL-7 receptor, could be used to identify memory precursor effector cells (MPECs) that display increased ability to form long-lived memory cells and short-lived effector cells (SLECs) that are poor at giving rise to long-lived memory cells (65). Additional studies suggested that expression of costimulatory molecule CD27, could identify effector cells that were more likely to survive contraction (66). Later, expression of KLRG1 in addition to CD127 was used to identify SLECs (CD127^−^/KLRG1^+^) and MPECs (CD127^+^/KLRG1^−^) (38). However, despite promoting survival of effector CD8 T cells to memory, CD127 expression and IL-7 signaling are not sufficient to drive formation of memory CD8 T cells, as forced expression of CD127 expression did not rescue survival of KLRG1^hi^ cells into memory (67). In addition, priming of naïve CD8 T cells in low inflammatory environment (ex. peptide-DC immunization) will generate CD127 expressing effector CD8 T cells prone to vigorous contraction (25, 68). Of note, displaying the expression pattern of markers used to identify SLECs (CD127^−^/KLRG1^+^) does not absolutely preclude long-term memory formation, as a small percentage of CD127^−^/KLRG1^+^ cells can be found months after infection (69, 70). Thus, the expression pattern of CD127 and CD27 on effector CD8 T cells mark cells with differential probability to survive contraction, but also highlights the notion that those markers cannot be used with certainty to predict effector cells that will become long-lived CD8 T cell memory.

### Tissue resident memory (Trm)

Tissue surveillance was a function first ascribed to circulating Tem cells (71). However, elegant parabiosis experiments have made it clear that some cells within tissues are not circulating, but are permanent residents (50, 51, 72). Efforts to identify tissue resident memory T cells (Trm) have shown that, unlike circulating cells, Trm cells are not labeled by intravenous injection of antibodies (73), with the noted exception of liver Trm cells, which are exposed to the circulation (74). In addition to tissue residence, Trm cells often are identified based on expression of integrins CD103 and CD49a, which aid in tissue entry (52, 53), and CD69, which promotes tissue retention (54). However, expression of these proteins can vary depending on tissue of residence. Trm cells are also described as expressing CXCR3 and lacking expression of KLRG1, CCR7, and CD62L, and having intermediate or low expression of Cx3Cr1 (33, 43, 52). However, it was recently reported that cells that previously expressed KLRG1 can form Trm cells, and such ex-KLRG1 cells may delineate heterogeneity within the Trm population, as they express higher levels of granzymeB than Trm cells that never expressed KLRG1 (75). Responsiveness to TGF-β in most cases is necessary for Trm development (55, 76), and expression of transcription factors play an important role in promoting TGF-β responsiveness and retention of Trm cells within tissues. Transcriptionally, Trm cells are noted for reduced expression of KLF2 and Eomes (55, 56), low expression of T-bet and TCF1 (55, 57), and elevated expression of Hobit and Blimp1 (57). Trm-mediated protection in peripheral tissues is primarily mediated through sensing and alarm functions. This requires Ag recognition and IFN-γ production by Trm cells, results in global modification of gene expression within inflamed tissues and increased expression of chemokine ligands, and promotes recruitment and effector functions of cells of the innate and adaptive immune system (58, 77–79). Trm cells provide protection against diverse microorganisms in an array of tissues including the lungs (33), salivary glands (80, 81), female reproductive tract (58, 78), skin (28), and liver (74). Because of this, attempts to generate Trm cells with site-directed vaccinations are being pursued.

### Tcm, Tem, and peripheral memory (Tpm) subsets based upon Cx3Cr1 expression

Recently, characterization of Tem and Tcm subsets was further refined, and an additional memory subset was described following the identification of Cx3Cr1^int^ peripheral memory (Tpm) T cells (43). Staining for CD27 or CXCR3 and Cx3Cr1 (fractalkine receptor) permits identification of Cx3Cr1^−^, Cx3Cr1^int^, and Cx3Cr1^hi^ populations at a memory time point. Cx3Cr1^hi^ cells do not migrate toward CCR7 ligand CCL19, do not re-express CD62L, are absent in lymph nodes but abundant in the circulation and tissues, proliferate and produce IL-2 poorly in response to Ag, and are efficient killers of target cells. These characteristics overlap with Tem cells and imply that expression of Cx3Cr1 may identify a homogeneous Tem population. Conversely, Cx3Cr1^−^ and Cx3Cr1^int^ populations are found in the lymph nodes and migrate in response to CCL19, suggesting that expression of Cx3Cr1 can be used to distinguish two populations among cells that would be defined as Tcm cells. Cx3Cr1^−^ cells display characteristics of classically defined Tcm cells in that they are more prevalent in lymph nodes, re-acquire CD62L faster and to a greater extent, and are better producers of IL-2 but less cytotoxic than Cx3Cr1^int^ cells. Therefore, Cx3Cr1 may allow identification of a more homogeneous population of Tcm cells. While the majority of Cx3Cr1^−^ cells express CD62L 1 year after infection, approximately half of Cx3Cr1^int^ cells express CD62L, and formation of Cx3Cr1^int^ cells is reduced but not eliminated in T-bet deficient mice, suggesting further heterogeneity within the Tpm population. This distinction may be important, as a large percentage of inflationary memory CD8 T cells in mice and humans generated in response to adenovirus-vectored vaccines or natural cytomegalovirus (CMV) infection are Cx3Cr1^int^ (82), and it was suggested that a CD62L^hi^/Cx3Cr1^+^ population within the lymph nodes is important in providing protection against chronic infection (61). Importantly, Tem, Tcm, and Tpm populations identified based on Cx3Cr1 expression display different migratory patterns (43). Contrary to previous descriptions as tissue surveyors, Tem cells were excluded from tissues, but were highly represented in the circulation. Instead, the tissue surveyor role was ascribed to Tpm cells, which could traffic to the tissues and return to lymph nodes via afferent lymphatics. These data call for a refinement to the hypothesis of the role of memory CD8 T cell subsets in providing host protection and suggest that immuno-surveillance is mediated by discrete actions of Tem cells, which are cytotoxic and present in the circulation and can be easily recruited to sites of inflammation; Tcm cells, which are centrally localized within lymph nodes and are highly proliferative following Ag re-encounter; Trm cells, which respond to infections arising in peripheral tissues and proliferate and recruit other immune cells following infection; and Tpm cells, which survey peripheral tissues and may be important for mediating protection against chronic infections.

### Additional memory cell subset classifications

Classifications of memory CD8 T cells into, Tem, Tcm, Trm, and Tpm subsets informs our understanding of immuno-surveillance provided by CD8 T cells of discrete functionality, but it does not capture the complete diversity within the memory CD8 T cell pool. Additional subsets have been described based upon expression of CD27 and CD43, a glycosylated form of sialic acid (32, 62). CD27^lo^/CD43^lo^ memory cells are KLRG1^hi^, CD127^lo^, T-bet^hi^, and Eomes^lo^ (32), an expression pattern that overlaps with, but is not identical to either Tem or Trm cells. Importantly, CD27^lo^/CD43^lo^ memory cells provide superior protection against Sendai virus and *Listeria monocytogenes* infection, perhaps due to an ability to localize to tissues. Thus, Tem, Tcm, Trm, and Tpm classification does not completely capture memory CD8 T cell diversity. Examination of additional markers may improve resolution of existing subsets and expand the number of identifiable subsets in the future, and lead to an improved understanding of memory CD8 T cell-mediated immuno-surveillance.

## Effects of time and ag-encounters on memory CD8T cell pool composition

### Time

Long-lived hosts can re-encounter pathogens at any time, and studies have indicated that the phenotype, function, and protective abilities of Ag-specific memory CD8 T cells change with time following infection. The percentage of circulating pathogen-specific memory CD8 T cells expressing CD27 and CD62L increases with time after infection, (30, 83–85), and the percentage expressing Cx3Cr1 decreases (43, 75), indicating that representation of Tcm cells among pathogen-specific memory CD8 T cells increases with time after infection. As would be expected of Tcm cells, aged or late memory cells proliferate and produce IL-2 to a greater extent than early memory cells in response to Ag (69, 70, 86, 87), and provide enhanced protection against chronic viral infection (69, 70). Changes observed in late memory cells extended beyond phenotype and functions normally attributed to Tcm cells, including increased ability to up-regulate expression of FasL and CD40L and to produce XCL1; decreased expression of many cytokine and chemokine receptors including IL-10R, components of IL-12R and IL-18R, CCR2, and CCR5; and decreased ability to produce IFN-g in response to inflammatory cues in the absence of cognate antigen recognition (bystander activation) (70, 88). Strikingly, phenotypic heterogeneity of Tcm cells was diminished with time after infection, and progressive changes in transcriptomic, phenotypic, and metabolic profiles of Tcm cells indicated an improved proliferative capacity of Tcm cells with time after infection, leading to an increased ability to provide protection against LCMV-clone 13 infection (69). In contrast, the percentage of CD62L^lo^ cells decreases with time after infection (69, 70, 83, 84), indicating decreased representation of Tem cells. Of note, the CD62Llo subset is comprised of not only functional, IFN-g producing Tem but also of recently identified T death intermediate memory (Tdim) cells (89). Tdim arise from the process of memory CD8 T cell homeostatic proliferation, are non-functional, and are destined to die, (89) and their representation increases among CD62Llo Tem subset with time after infection (69).

**Figure 1:**
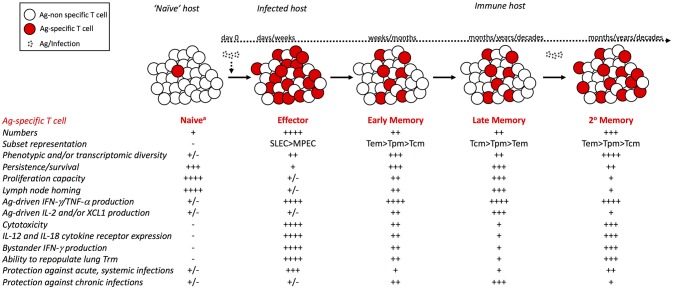
Phenotypic and functional changes within the circulating Ag-specific CD8 T cell pool with time after infection and with additional Ag encounters. Following infection or vaccination, rare naïve CD8 T cells that recognize their cognate Ag robustly proliferate and give rise to an effector CD8 T cell population. Following contraction of the effector pool, memory CD8 T cells are stably maintained at numbers greater than the naïve pool. Upon re-infection or booster vaccination, primary memory CD8 T cells proliferate and generate a secondary effector and memory CD8 T cell pool that is larger in size than the primary memory pool. Properties of cells comprising the Ag-specific CD8 T cell pool, including expression of phenotypic markers and subset representation, ability to traffic to and localize within tissues, ability to execute effector functions, and ability to provide protection against infection with diverse pathogens differ between naïve, effector, and memory CD8 T cells, and among memory CD8 T cells of different age relative to initial Ag-encounter and of different number of Ag-encounters. – symbols indicate reduced quantity or ability, while + symbols indicate increased quantity or ability. ^a^Virtual and/or innate memory cells within the naïve CD8 T cell pool are not considered here.

Like Tem cells, numbers of Tpm cells decrease initially after infection, but following an initial period of decline, they are maintained at stable numbers (43). However, the percentage of CD62L^hi^ Tpm cells increases with time after infection. Few studies have examined the properties of long-term Trm cells, and it is unclear how the functions of Trm cells are affected by time. Trm cells in the skin persist for >300 days after infection and are long-lived (28). However, influenza-specific Trm cells in the lungs are shorter-lived (90) and require replenishment by circulating CD62Llo memory cells (91). Together, these studies indicate that with time after infection, the circulating Ag-specific memory CD8 T cell population is comprised of a more homogeneous population of Tcm cells with enhanced proliferative capacity, which impacts host CD8 T cell-mediated protection against infection (Figure 1).

### Ag-encounters

Hosts are often exposed to the same pathogens throughout life, and prime-boost vaccination protocols intended to increase memory cell numbers result in memory CD8 T cells that have encountered Ag multiple times. Initial experiments utilizing adoptive transfer of purified TCR transgenic or tetramer positive memory cells of known Ag-encounter history showed that additional Ag-encounters result in decreased representation of circulating memory CD8 T cells expressing CD62L, CD27, and CD127, and increased representation of cells expressing KLRG1 and GranzymeB (83, 84) (Figure 1). Phenotype of secondary memory CD8 T cells is also greatly impacted by systemic inflammation elicited during the infection/vaccination (92). Successive Ag-encounters also result in stepwise changes in transcriptomic signature of memory CD8 T cells, but not in progressive enrichment in Tem associated genes (93), suggesting that additional Ag-encounters result in generation of a more transcriptionally diverse Tem population. Differences in memory CD8 T cell composition and function with additional Ag-encounters translate to differential ability to provide protection against re-infection, with memory CD8 T cells that have encountered Ag multiple times being more protective against re-infection with *Listeria monocytogenes*, LCMV-Armstrong, and Vaccinia Virus, and less protective against re-infection with MHV and LCMV-clone 13 (29). However, recurrent homologous boosting preserves numerical stability and increases phenotypic and functional complexity of the memory CD8 T cell pool (94), and sequential heterologous infection results in a pool of Ag-specific memory CD8 T cells with a phenotypic profile reflective of Tcm cells that are metabolically fit, proliferate robustly following re-infection, and provide protection against LCMV-clone 13 (34). Although homologous and heterologous infection strategies likely result in mixed memory populations with cells that have encountered Ag a different number of times, and thus are not reflective of pure memory populations of known number of Ag-encounters, they may more accurately reflect sequential infections in humans.

Recent examinations of Trm cells that re-encounter Ag have shown that Trm cells proliferate within tissues and contribute to formation of secondary Trm cells (95, 96), and can migrate to, and form Trm populations within tissue-draining lymph nodes (97). Importantly, although Ag-exposure history defines CD8 T cell dynamics and protection during localized pulmonary infections (98) lung Trm derived from repeatedly stimulated influenza-specific circulatory memory CD8 T cells exhibit extended durability and protective heterosubtypic immunity relative to primary lung Trm (99). Parabiosis studies reveal that repeated antigen encounters resulted in generation of long-lasting circulating effector memory (Tem) cells that maintained their ability to be recruited to the lung parenchyma and converted to Trm (99). Thus, successive Ag-encounters also results in diversification of the Trm subset, which impacts their ability to provide protection against subsequent infections arising at peripheral locations.

## Memory CD8T cell heterogeneity and subsets in humans

Humans are exposed to an array of infections throughout life and often re-encounter the same infection. Additionally, it is often difficult to determine precisely when infection was encountered, and due to obvious difficulties in acquiring human tissue samples, the majority of human studies rely on analysis of CD8 T cells in peripheral blood. These considerations have presented difficulties for examining memory CD8 T cells of known age relative to Ag-encounter and of known number of Ag-encounters in humans, but recent studies have provided insight into subset composition and heterogeneity present within the memory CD8 T cell population of humans. Most human studies rely on analysis of bulk CD8 T cell populations, and similar to mice, Tcm and Tem subsets can be identified in humans, along with a terminally differentiated subset that expresses CD45RA (Temra). Initial characterization of these subsets was based on expression of CD45RA and CD27 (44), while later studies distinguished Tem and Tcm subsets based upon expression of CCR7 (37), and staining for CD45RA and either CD27 or CCR7 identifies naïve (CD45RA^+^/CD27^+^/CCR7^+^), Tem (CD45RA^−^/CD27^−^/CCR7^−^), Tcm (CD45RA^−^/CD27^+^/CCR7^+^), and Temra (CD45RA^+^/CD27^−^/CCR7^−^) CD8 T cells. Memory CD8 T cells of distinct phenotypes accumulate with age, and accumulation of Temra cells in humans is influenced by chronic infections, such as CMV (100, 101).

Recent studies with organ donors of diverse ages have provided some clarity on the compartmentalization of human memory subsets, describing large populations of Trm cells, and regional surveillance by Tem, Tcm, and Temra cells that varied depending on the tissue and were not reflective of subset representation within the blood (59, 60). As in mice, protection against infection is likely mediated by cells of discrete phenotype and function that cannot be fully described based upon classification of Tem, Tcm, Temra, and Trm subsets. Recently, human memory subsets were described based on Cx3Cr1 expression, and a highly cytotoxic Cx3Cr1^+^/CD62L^+^ subset that resides within the lymph node was suggested to be important for mediating protection against chronic infections including CMV (61).

Due to the endemic nature of most pathogens that humans are vaccinated against, it is difficult to examine Ag-specific memory CD8 cells of known age relative to initial activation and number of Ag-encounters in humans. However, experiments with vaccines for small pox and yellow fever virus (YFV), which are not endemic within the United States, have allowed for examination of primary memory CD8 T cells of known age relative to initial Ag-encounter. Expression of CD45RA, CD127, and CCR7 on Ag-specific memory CD8 T cells increased, while expression of perforin and granzymeB decreased with time after infection, suggesting that similar to mice, representation of Tcm cells within the Ag-specific human memory CD8 T cell population increases with time after infection (22, 102). However, while cytotoxic functions of memory CD8 T cells appeared to decrease with time after infection based upon expression of perforin and granzymeB at steady state, memory cells retained open chromatin configurations at locations relevant for cytotoxicity and cytokine production, suggesting that genes encoding for effector functions are readily open for transcription following Ag re-encounter (19, 22). Recent reports in mice have also shown dynamic epigenetic regulation of genes driving CD8 T cell localization and function during differing differentiation states (23). DNA methylation patterns of *Sell* (the gene encoding CD62L) were restrictive in effector cells, but demethylated in naïve and memory cells. Conversely, *GzmB* (the gene encoding granzymeB) displayed restrictive methylation patterns in naïve cells, but were demethylated in effector cells and memory precursor cells (23). These recent studies have indicated that, as in mice, the memory CD8 T cell pool in humans is composed of subsets with discrete functionalities, and subset composition likely impacts host immuno-surveillance in response to diverse pathogens.

## CD8T cell responses in alternative mouse models

Human studies have pointed to many similarities between CD8 T cell responses in mice and humans. However, differences that exist between mice and humans may limit translational value of mouse research. Recent efforts to extend mouse models outside of traditional inbred mice housed in specific pathogen free (SPF) facilities have provided valuable insight into CD8 T cell biology. In contrast to the CD8 T cell compartment of SPF laboratory mice, which consists primarily of naïve T cells and is similar to that of a neonatal human, sequential infections with common pathogens or co-housing laboratory mice with wild/pet store (“dirty”) mice generates a CD8 T cell compartment that is similar to adult humans and is comprised of a large number of Ag-experienced CD8 T cells with increased representation of cells in peripheral tissues (103). Additionally, a greater percentage of memory phenotype CD8 T cells of “dirty mice” displayed phenotypic markers expressed by Tem cells and were more cytolytic than memory phenotype cells of SPF laboratory mice (103). *De novo* immune responses in “dirty mice” resulted in reduced Ab production compared to SPF mice, and displayed transcriptional similarities to adult human blood in contrast to SPF mice, which displayed transcriptional similarities to neonatal humans (103, 104). Furthermore, memory CD8 T cells of “dirty mice” that developed following infection with *Listeria monocytogenes* were more skewed toward a SLEC phenotype compared to SPF mice, and “dirty mice” were better protected against infection with *Listeria* and *Plasmodium berghei* (103). These studies suggest that “dirty mice” may more closely model the immune system of adult humans, and that history of pathogen exposure shapes the immune system and impacts phenotype of memory CD8 T cells generated and protection provided following *de novo* infection. Future studies should more closely examine the innate and adaptive immune factors that are shaped following sequential infection with unrelated pathogens, and how these interact to generate a qualitatively different CD8 T cell response following *de novo* infection.

Additional insight has been gained from studies utilizing outbred mice to model genetic diversity present in the human population. Ag-driven changes in expression of CD8α and CD11a have been used as “surrogate activation markers” approach to track pathogen-specific CD8 T cell responses to infection in outbred mice without a priori knowledge of MHC class I restriction and/or specific epitopes (105). Data revealed that compared to uniformity in size of the effector and memory responses generated in inbred mice, magnitude of effector and memory CD8 T cell responses are highly variable in individual outbred mice (105, 106). Furthermore, while memory CD8 T cells in inbred mice progressed linearly from a Tem to Tcm phenotype with time, percentages of memory cells expressing Tcm markers (CD62L^hi^, CD27^hi^, CD127^hi^, KLRG-1^lo^) did not increase or increased very slowly with time after infection in some outbred mice (106). Importantly, differences in CD8 T cell responses generated to a primary infection in outbred mice led to differences in CD8 T cell-mediated protection provided against a secondary infection, and degree of protection did not always correlate with size of the memory CD8 T cell pool prior to secondary infection (105, 106). These studies suggest that vaccine strategies that generate a memory CD8 T cell pool of sufficient size and quality to provide protection against re-infection in inbred mice may not generate a protective memory CD8 T cell response in all outbred mice, a finding that has direct relevance to the outbred human population.

Differences in memory CD8 T cell response size and phenotype following infection in individual outbred mice could have been caused by a number of immunologic factors including differences in cells of the innate compartment or differences in Th bias of the CD4 T cell compartment. However, underlying causes for divergent CD8 T cell-mediated immune outcomes were unable to be fully explored in the studies discussed due to a lack of tools available for study in outbred mice. Collaborative cross mice, a recombinant inbred panel of mice that displays vast genetic diversity due to unique inheritance from eight founder strains (107, 108), and diversity outbred mice, which are generated by outcrossing collaborative cross strains at various stages of the inbreeding process (109), may prove to be useful models for deciphering the answers to this question. Studies with collaborative cross mice have revealed a range of immune cell composition, phenotype, and function among strains prior to infection that is more representative of the human population (110), and post-infection outcomes relevant to the human population that are not observed in traditional inbred mice (111). Genetic tools uniquely suited for collaborative cross mice, including quantitative trait locus mapping (QTL) (112), may provide additional insight into factors underlying divergent memory CD8 T cell outcomes in genetically diverse organisms, and how memory CD8 T cells of diverse phenotype and function arise and participate in immune-mediated protection against re-infection.

## Conclusion

Protection against diverse pathogens that have evolved for unique interactions with hosts, different points of host entry, and colonization and replication within particular host cells requires a diverse and adaptable immune system. Heterogeneous memory CD8 T cells that can persist in and localize to different areas within the host, and that are functionally adapted to respond in discrete ways within their host niche, contribute to the diversity and adaptability needed to efficiently provide host immuno-surveillance. The effects of time following infection and additional Ag encounters further shape diversity of the memory CD8 T cell pool, which impacts efficacy of CD8 T cell-mediated protection against re-infection. Efforts to subset memory CD8 T cells have informed our knowledge of how CD8 T cells with discrete functionalities contribute to host immuno-surveillance against diverse microbial pathogens, and improved animal models that more accurately reflect the human immune system may improve our understanding of the origins and functions of memory CD8 T cells of diverse phenotype and improve translational value of current animal studies.

## Author contributions

All authors listed have made a substantial, direct and intellectual contribution to the work, and approved it for publication.

### Conflict of interest statement

The authors declare that the research was conducted in the absence of any commercial or financial relationships that could be construed as a potential conflict of interest.
